# The Central Association (in Austria) for the Erection of Public Slaughter-houses

**Published:** 1897-11

**Authors:** 


The Central Association (in Austria) for the Erection of Public Slaughter-houses.—At the session of the Central Association, October 16, 1896, an important report was made in reference to the veterinary sanitary police. It affected the question of public slaughter-houses, and was first brought up by the Veterinary Association of Mäluen. Herr Postolka, as chairman of the committee, read the report, which was adopted, with a few changes, on the second reading. The petition was then handed in to the Imperial Department of the Interior, with the hope that it would produce a prompt result.

The petition points out the fact that, in spite of meat-inspection, a great deal of a very doubtful character is still presented for sale, because the districts are so large and the inspection is often intrusted to general practitioners who cannot cover the whole district thoroughly, and through the press of their work have not time to keep pace with the rapidly developing science of bacteriology. As the best means of avoiding these evils, it prays the government to erect public slaughter-houses in the principal communities, and enact laws that all cattle shall be slaughtered in them. A veterinarian thoroughly practised in meat-inspection could be constantly on hand, and thus in the most economical manner the best results could be obtained.—*Idem.*

